# Application of Remote Sensing Data for Locust Research and Management—A Review

**DOI:** 10.3390/insects12030233

**Published:** 2021-03-09

**Authors:** Igor Klein, Natascha Oppelt, Claudia Kuenzer

**Affiliations:** 1 German Remote Sensing Data Center (DFD), German Aerospace Center (DLR), 82234 Wessling, Germany; Claudia.kuenzer@dlr.de; 2 Department of Geography, Kiel University, 24118 Kiel, Germany; Oppelt@geographie.uni-kiel.de; 3 Institute of Geography and Geology, University Wuerzburg, 97074 Wuerzburg, Germany

**Keywords:** locust monitoring, locust outbreak, remote sensing, locust habitat, locust pest

## Abstract

**Simple Summary:**

Locust outbreaks around the world regularly affect vast areas and millions of people. Mapping and monitoring locust habitats, as well as prediction of locust outbreaks is essential to minimize the damage on crops and pasture. In this context, remote sensing has become one of the most important data sources for effective locust management. This review paper summarizes remote sensing-based studies for locust management and research over the past four decades and reveals progress made and gaps for further research. We quantify which locust species, regions of interest, sensor data and variables were mainly used and which thematic foci were of interest. Our review shows that most studies were conducted for the desert locust, the migratory locust and Australian plague locust and corresponding areas of interest. Remote sensing studies for other destructive locust species are rather rare. Most studies utilized data from optical sensors to derive NDVI and land cover for mapping and monitoring the locust habitats. Furthermore, temperature, precipitation and soil moisture are derived from thermal infrared, passive and active radar sensors. Applications of the European Sentinel fleet, entire Landsat archive or very-high-spatial-resolution data are rare. Implementing new methods (e.g., data fusion) and additional data sources could provide new insights for locust research and management.

**Abstract:**

Recently, locust outbreaks around the world have destroyed agricultural and natural vegetation and caused massive damage endangering food security. Unusual heavy rainfalls in habitats of the desert locust (*Schistocerca gregaria*) and lack of monitoring due to political conflicts or inaccessibility of those habitats lead to massive desert locust outbreaks and swarms migrating over the Arabian Peninsula, East Africa, India and Pakistan. At the same time, swarms of the Moroccan locust (*Dociostaurus maroccanus*) in some Central Asian countries and swarms of the Italian locust (*Calliptamus italicus*) in Russia and China destroyed crops despite developed and ongoing monitoring and control measurements. These recent events underline that the risk and damage caused by locust pests is as present as ever and affects 100 million of human lives despite technical progress in locust monitoring, prediction and control approaches. Remote sensing has become one of the most important data sources in locust management. Since the 1980s, remote sensing data and applications have accompanied many locust management activities and contributed to an improved and more effective control of locust outbreaks and plagues. Recently, open-access remote sensing data archives as well as progress in cloud computing provide unprecedented opportunity for remote sensing-based locust management and research. Additionally, unmanned aerial vehicle (UAV) systems bring up new prospects for a more effective and faster locust control. Nevertheless, the full capacity of available remote sensing applications and possibilities have not been exploited yet. This review paper provides a comprehensive and quantitative overview of international research articles focusing on remote sensing application for locust management and research. We reviewed 110 articles published over the last four decades, and categorized them into different aspects and main research topics to summarize achievements and gaps for further research and application development. The results reveal a strong focus on three species—the desert locust, the migratory locust (*Locusta migratoria*), and the Australian plague locust (*Chortoicetes terminifera*)—and corresponding regions of interest. There is still a lack of international studies for other pest species such as the Italian locust, the Moroccan locust, the Central American locust (*Schistocerca piceifrons*), the South American locust (*Schistocerca cancellata*), the brown locust (*Locustana pardalina*) and the red locust (*Nomadacris septemfasciata*). In terms of applied sensors, most studies utilized Advanced Very-High-Resolution Radiometer (AVHRR), Satellite Pour l’Observation de la Terre VEGETATION (SPOT-VGT), Moderate-Resolution Imaging Spectroradiometer (MODIS) as well as Landsat data focusing mainly on vegetation monitoring or land cover mapping. Application of geomorphological metrics as well as radar-based soil moisture data is comparably rare despite previous acknowledgement of their importance for locust outbreaks. Despite great advance and usage of available remote sensing resources, we identify several gaps and potential for future research to further improve the understanding and capacities of the use of remote sensing in supporting locust outbreak- research and management.

## 1. Introduction

Locust and grasshopper pests have been destroying agriculture and affecting human lives by causing major food security challenges since ancient times and serious outbreaks are documented both in historical sources and modern literature [1,2,3,4]. There are approximately one dozen serious pest locust and grasshopper species, which are capable of migrating great distances and are destructive to crops, pastures and other green vegetation during their gregarious phase [5,6]. Locusts differ from other insects because their population can grow rapidly, forming dense bands and swarms [4]. In the solitarious phase, locusts are an important part of ecosystems. However, a change in environmental conditions and growth in population may initiate the gregarious phase, which can lead to an outbreak [4]. Furthermore, locust population dynamics are also influenced by land management [7]. For locust phase polyphenism and population density research, we refer the reader to [8,9,10,11].

One of the most destructive species, the desert locust (*Schistocerca gregaria*), is responsible for the most dramatic and sudden outbreaks and plagues in the 20th and 21st centuries [4,12]. Low populations of the desert locust are usually present at any time across a vast recession area of 16 million km^2^, stretching from West Africa to Southwest Asia [13]. Migrating downwind, the desert locust breed sequentially where winter, spring and summer rains are falling [14]. Warm weather conditions and unusual heavy rainfalls combined with a lack of monitoring created perfect conditions for the recent 2019/2020 outbreak, which was evident in large occupied areas across East African countries, the Arabian Peninsula, Pakistan and India [15,16]. Apart from desert locust outbreaks, there were local outbreak occurrences of the Moroccan locust (*Dociostaurus maroccanus*) in parts of Central Asia, the Italian locust (*Calliptamus italicus*) in parts of East Russia, the South American locust (*Schistocerca cancellata*) in parts of Paraguay and Argentina, the African migratory locust (*Locusta migratoria migratorioides*) in Botswana, Namibia, Zambia and Zimbabwe as well as Yellow-spined bamboo locust (*Ceracris kiangsu*) in parts of Vietnam, Laos and China [17]. Furthermore, an unexpected Moroccan locust outbreak during summer 2019 and 2020 destroyed several thousand hectares of crops in Sardinia, Italy [18]. These recent large-scale as well as local outbreak events of different locust species around the world underline the actual presence of locust pest risk for food security, their destructive effects and the importance of functioning locust management services.

**Figure 1:**
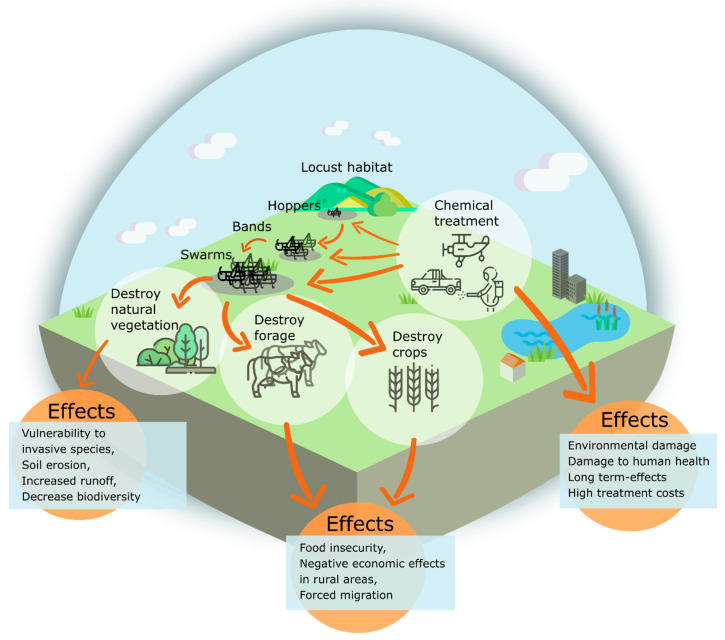
Schematic sketch of locust interaction during the gregarious phase (outbreak) with the natural environment, agriculture and human settlements. Information extracted from [3,4,20].

Outbreaks of locust and grasshopper are either chronic (e.g., grasshoppers in the African Sahel and grasshoppers/locusts in China) or episodic, with alternating periods of invasion and recession (e.g., the Australian plague locust and the desert locust) [4]. Locust outbreaks have many negative effects on land management, food security and the natural environment, ranging from total damage of crops and grazing fields to negative effects from control measurements when using insecticides. In Figure 1, we summarize general effects of locust outbreaks. In particular, the damage to crops and chemical contamination caused by control measurements have short- to long-term negative impacts [4,19].

**Figure 2:**
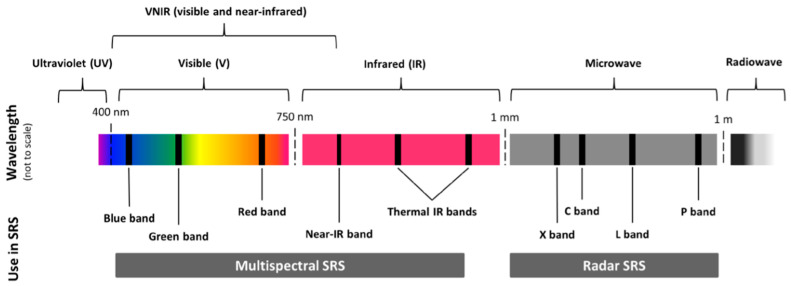
Electromagnetic radiation spectrum with bands used in satellite remote sensing (SRS) (from [25]).

Due to the size of the impact, locust management and control are essential. Locust management is complex and requires a multi-disciplinary approach including entomology, biology, and ecology, with aspects of spatial distribution modelling, climate analysis, weather prediction, organism behavior and interaction with other species (e.g., birds and grazing sheep), control using chemical insecticides or bio-agents as well as remote sensing applications. The latter has become one of the most important sources providing valuable information within locust management. Meanwhile, there is a wide range of existing passive (employ natural sources of energy) and active (emit a controlled beam of energy and detect the amount of energy reflected back to the sensor) Earth Observation (EO) sensor systems. For a detailed introduction to remote sensing, we refer the reader to [21,22,23]. The most important sensor characteristics are the spectral resolution (number of spectral bands), spatial resolution (smallest unit-area indicating the minimum size of objects that can be detected), temporal resolution (time between two observations of one and the same location) and spatial coverage (total area covered by one image). For this review, important sensor types can be categorized into optical sensors (covering visible, near infrared (NIR) and short-wave infrared (SWIR) spectrum) and sensors covering thermal infrared (TIR). Spaceborne radar (RAdio Detection and Ranging) remote sensing includes passive and active systems. While active sensors are usually characterized by higher-spatial-resolution, passive microwave sensors operate on coarser spatial resolution [24]. The electromagnetic radiation spectrum with important bands used in satellite remote sensing (SRS) is shown in Figure 2.

Remote sensing-based research and case study applications were important drivers to improve our understanding of locust-relevant ecological and environmental conditions. Since the 1980s, information acquired from remote sensing data has accompanied many locust management activities and contributed to improved and more effective control of locust outbreaks and plagues around the world. Nevertheless, locust outbreaks still cause devastation and hunger, despite technological progress and improvement in monitoring and control. One of the reasons is the ineffective monitoring, management or population control in some locust habitats, e.g., due to lack of available resources and technology [15]. Environmental changes (e.g., land use alterations) and weather variability within the locust habitats can create optimal conditions for locust breeding, which needs to be realized and control undertaken in time. Otherwise, such changes may lead to increased population, causing a transition from the solitarious phase to the gregarious phase and therefore initiate a locust outbreak. Therefore, continuous monitoring during the solitarious phase is essential. Apart from short- to mid-term variability of important ecological variables, the effect of climate change is also considered to be a factor for more frequent and severe outbreaks [26,27,28]. 

**Figure 3:**
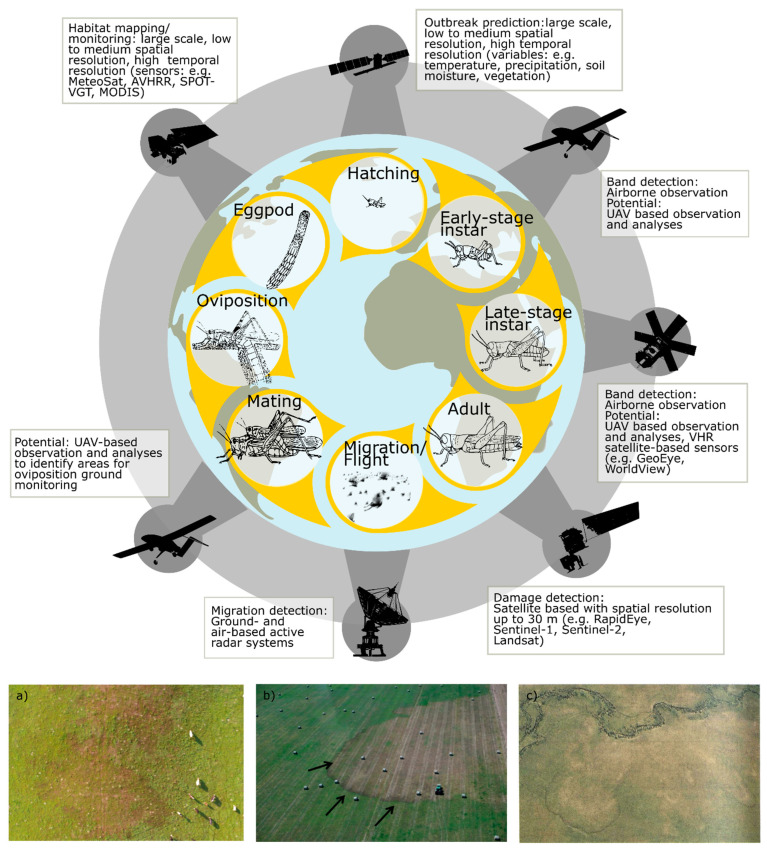
Upper: Representative life cycle of locust and grasshopper species including critical phases for locust management and where remote sensing can provide support and provide data. (**a**) Red, green, blue (RGB) image taken by a UAV from 80 m height with visible vegetation damage caused by early stage of the Moroccan locust (South Kazakhstan, April 2019). (**b**) Aerial image of bands of the Australian plague locust and visible caused damage (source: Figure 60 from [37], photos from Victorian Government Agriculture Department). (**c**) Bands of the Australian plague locust and damaged vegetation visible in airplane-taken RGB image from 400 m height (source: Figure 2 from [38]).

The Food and Agriculture Organization (FAO) has been successfully introducing standardized monitoring methods and data collection when remote sensing data and applications play an essential role. Remote sensing data related to locust outbreaks was first introduced by Pedgley [29] and Hielkema [30] and was later implemented in FAO operative desert Locust Information Service (DLIS). Hielkema at al. [31] and Hielkema and Snijders [32] focused on Meteosat cloud imagery to estimate rainfall, and on Landsat and AVHRR-based estimation of vegetation development. The Australian Plague Locust Commission (APLC) is another organization successfully utilizing remote sensing data to support locust management [33,34]. Since then, FAO and APLC and different research projects have contributed to a steady progress in implementing remote sensing-based products. In general, remote sensing can provide different kinds of information at different critical moments within the locust life cycle. Figure 3 represents a typical locust life cycle and sketches where remote sensing technologies have been applied in the past and present or have the potential for future applications. These applications can be summed up in following overarching topics:

- Mapping and monitoring the locust habitat state and environmental conditions which promote the transition process between the solitarious and gregarious phases.
- Prediction of hatching time and possible outbreaks based on historical information, present vegetation monitoring and weather forecast.
- Locust nymph bands and swarm monitoring with airborne or UAV-based sensors.
- Post outbreak crop and vegetation damage assessment.
- In addition to EO remote sensing, direct radar (X-band) observations of ‘migration in progress’ have been used for research on the migration systems of locusts and migratory grasshoppers, particularly for the Australian Plague locust and the Senegalese grasshopper [35]. Insect-monitoring radars (IMRs) are currently used to supplement existing survey and monitoring programs of the Australian Plague locust [36].

This review aims to provide a comprehensive and quantitative overview on ‘satellite-based’ remote sensing applications and research within critical phases for locust management. Due to high potential for locust management, as well as similar principles in image interpretation and processing, we also included UAV and airborne-based studies. We aim to summarize past and present developments and identify topics which still require further research and scientific attention. This review is structured as follows: in Section 2. Materials and Methods, we present the applied literature search and categorize different publication-specific aspects and thematic foci which are reviewed and presented separately. In Section 3. Results, we present the outcome for each aspect and summarize most important findings. In Section 4. Discussion, results are critically discussed, gaps and further potential are stated. In Section 5. Conclusion, we summarize and underline main findings.

## 2. Materials and Methods

**Figure 4:**
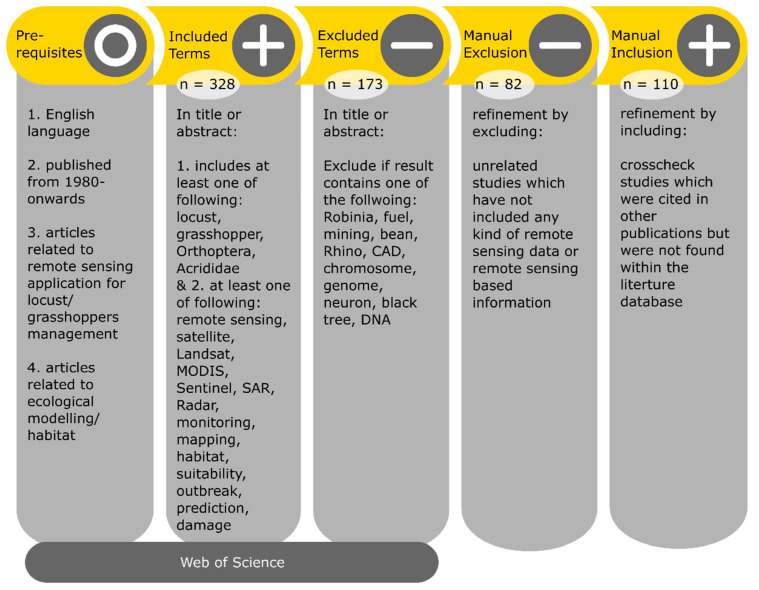
Workflow and literature searching criteria applied for this review.

Locust pest research and management cover several scientific disciplines. Therefore, potential articles cover a broad range of journals. For this review, we systematically reviewed 110 scientific publications including remote sensing applications which were published since 1980. The conducted literature search was based on the bibliographic digital database of Web of Science (last accessed on 15 December 2020) including Science Citation Index (SCI) journals and full-text conference contributions (Figure 4). For the literature search, we used specified terms and additional keywords including ‘locust’, ‘locust pest’, ‘locust plague’, ‘locust outbreak’ and ‘grasshopper’ in combination with ‘remote sensing’ or ‘satellite’, ‘UAV’, ‘airborne’ as well as ‘habitat’, ‘monitoring’, ‘prediction’, ‘control’, and ‘management’. This search query resulted in a very large number of research articles also including publications which are not related to locusts and grasshoppers (*Orthoptera: Acrididae*). Therefore, additional excluding keywords were applied. In a final step, we screened the resulting publications based on the following inclusion criteria which are relevant for this review:

- Articles are related to locust and grasshopper species (*Orthoptera: Acrididae*).
- Articles should be based or include EO, airborne or UAV data as one of the data sources.
- Articles investigated either locust/grasshopper habitat, presence, or outbreak prediction.
- Articles are related to locust/grasshopper ecological modelling or population distribution with EO-based input.
- Articles related to locust/grasshopper damage monitoring/mapping with EO.

The literature review workflow and number of studies for each step are summarized in Figure 4.

The total selected 110 studies were analyzed to extract relevant information for this review in two main aspects. The first aspect includes publication-specific information about “species of interest”, “region of interest”, “applied remote sensing sensor” and “derived variables from remote sensing data”. Additionally, we extracted involved authors’ affiliation to investigate where main research is based compared to regions of interest. The second aspect includes thematical foci which were categorized into “habitat mapping”, “habitat monitoring”, “forecast of hatching/outbreak”, “damage assessment” as well as “review and general articles” without a specific data analyzing part (Table 1). 

## 3. Results

### 3.1. Development over Time

**Figure 5:**
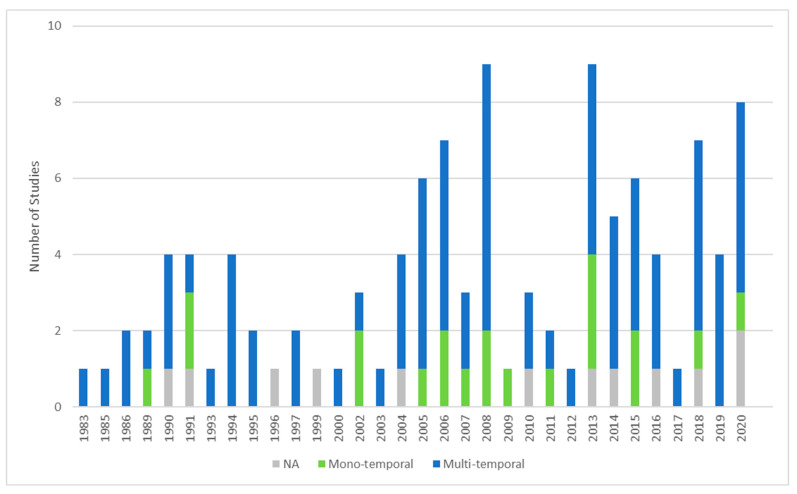
Total number of studies dealing with locust or grasshoppers applying remote sensing data (Mono-temporal = 18%, Multi-temporal = 71%, NA = 11%, see text for definitions of terms).

In this section, we recap the historical development of studies related to locust research and management applying remote sensing data (Figure 5). The first studies were published by Pedgley [29] and Hielkema [30] using Landsat Multi-Spectral Scanner (MSS) data to detect the presence of green vegetation in desert locust habitats in northwest Africa. After recognizing the potential of satellite imagery, the 1980s and 1990s were dominated by a few experimental studies and pioneer research on how remote sensing data analysis and application could be utilized to provide valuable information for locust management and to be implemented into operational services. Referring to locust plagues, Hielkema [39] introduced satellite remote sensing for desert locust habitat monitoring as “a new technology to an old problem”. McCulloch and Hunter [40], Bryceson and Wright [41], Bryceson and Bryceson et al. [33,42,43,44] investigated the usage of Landsat MSS imagery to identify and monitor habitats of the Australian plague locust. Tucker et al. [45] introduced the potential of AVHRR and Landsat datasets to forecast desert locust activity. Further feasibility studies followed for the Senegalese grasshopper (*Oedaleus senegalensis*) [46,47,48], the brown locust [49], and the Moroccan locust [50].

At the beginning of the new millennia, there was a slight increase in publications and a trend towards more specific studies related to outbreaks between 1999 and 2001 in Central Asia, Russia, China, Australia as well as desert locust outbreak in 2003–2005 in West Africa. This increase is visible in a first significant accumulation of studies from 2004 with the peak in 2008. The second peak of studies in 2013/2014 is related to a special issue “Advances in Remote Sensing Applications for Locust Habitat Monitoring and Management in the Journal of Applied Remote Sensing” with a total of 14 studies. The peaks in 2018 and 2020 can be related to an open source policy and accessibility of different satellite data archives and following new approaches (e.g., soil moisture and ecological niche modelling), as well as overall increased public and research interest and available funding probably related to recent severe outbreaks.

In general, it is clear that remote sensing application studies, at least those published in the English language, were rather rare until the start of the new millennium, mostly driven by research developments in collaboration between research centers and universities with FAO and APLC for monitoring and prediction service for the desert locust and the Australian plague locust. Afterwards, the academic interest involving EO data increased in the past two decades. Nevertheless, a significant development observed in other disciplines, e.g., related to new available EO data sources (e.g., Sentinel fleet) or opening long term archives (especially Landsat) is not evident. The observed accumulation of studies is related to locust outbreaks rather than technological advances and availability of remote sensing data. However, recent analysis related to soil moisture [51,52,53,54] as well as ecological niche modelling [5,55,56] based on several data sources were the focus of investigation and showed promising results.

In terms of the investigated temporal scale, 18% of all studies were conducted only for one image representing the conditions at the time of overfly (mono-temporal). A total of 71% of studies were conducted for several images representing several states at different time steps or temporal development (multi-temporal, see also Figure 5). Within multi-temporal studies, we can further discriminate between studies which applied multiple mono-temporal processing steps to mirror the state at these dates (28%), and studies applying time-series analyses (43%). Studies marked as “NA” (11%) are reviews and general articles without a specific data analysis part. 

**Figure 6:**
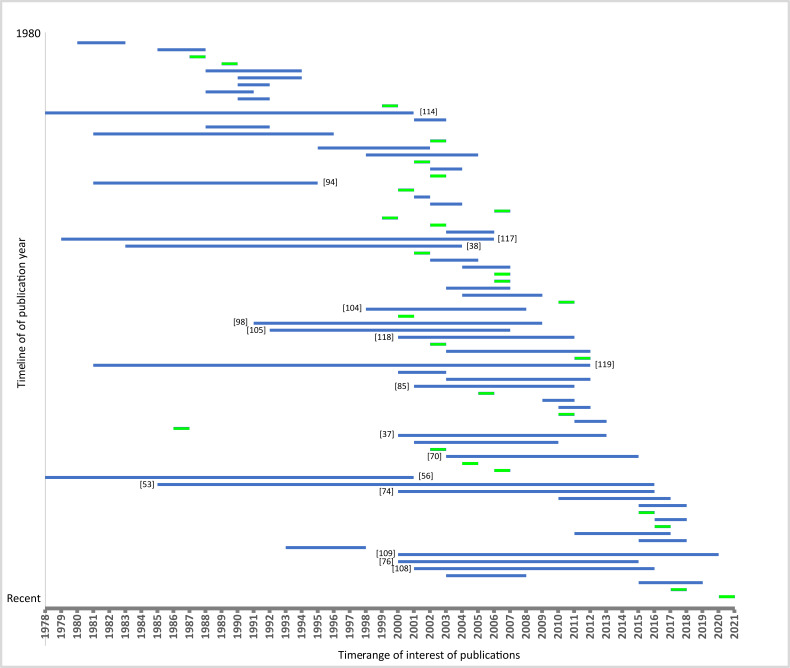
Temporal coverage of investigation within reviewed articles (green: mono-temporal studies, blue: multi-temporal studies; references indicate studies analyzing ten or more years).

Figure 6 shows the investigated time periods. It is obvious that most multi-temporal studies focus only on few years rather than longer time periods. In total, there are only 18 studies which cover at least ten or more years (added citation in Figure 6).

### 3.2. Publication-Specific Aspects

#### 3.2.1. Species of Interest

**Figure 7:**
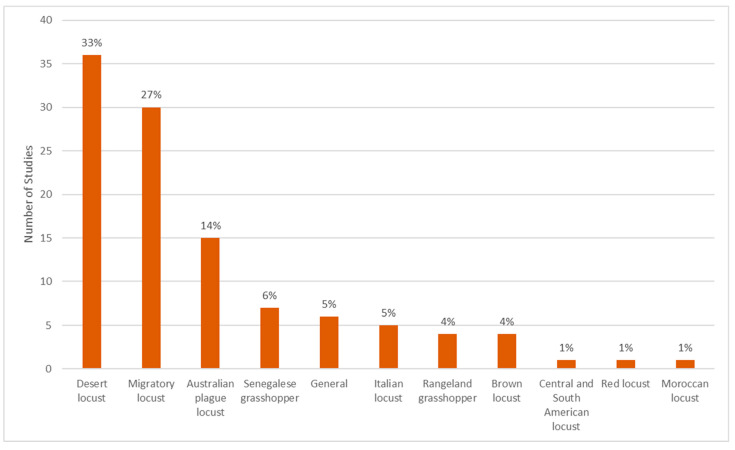
Total number of studies categorized by locust and grasshopper species. Note: the category migratory locust includes all subspecies, e.g., the Oriental, African and Asian migratory locusts.

Two species dominate the publications, i.e., the desert locust (33%) and the migratory locust (27%) (Figure 7). The migratory locust includes approximately ten subspecies which slightly differ biologically and morphologically, yet are characterized by similar ecological requirements [57]. Therefore, we consider this species as one overarching group. The third most investigated species is the Australian plague locust (14%). Few studies were found for the Senegalese grasshopper (6%), the Italian locust (5%), the brown locust (4%) and rangeland grasshoppers (e.g., *Heiroglyphus nigrorepletus, Oedaleus decorus asiaticus*, *Rhammatocerus schistocercoides*; 4%). Studies for other destructive species such as the Central and South American locusts (1%), the Moroccan locust (1%) and the red locust (1%) are rare. The category *General* (5%) does not focus on specific species but rather summarizes review papers including several species or general research which is relevant for more than one species (e.g., climate change).

#### 3.2.2. Area of Interest

**Figure 8:**
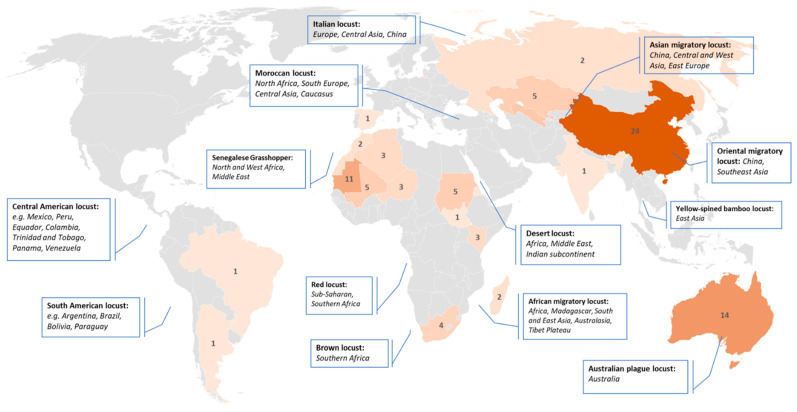
Regions of interest of reviewed studies. Comments indicate the most destructive locust species and their distribution [3,8].

In this section, we would like to pay attention to countries and regions of interest which were in focus of reviewed publications (Figure 8). Obviously, the area of interest is related to the species and its habitat distribution. Nevertheless, several species habitats cover large areas and invasion regions across several countries. For example, the countries of the Sahel region, especially Burkina Faso, Chad, Ethiopia, Eritrea, Mauritania, Mali, Niger, Nigeria, Senegal, Somalia, and Sudan are particularly susceptible to the desert locust [5]. In general, the desert locust breeds extensively in arid and semi-arid zones extending from West Africa through the Middle East to Southwest Asia including the Arabian Peninsula, Pakistan and India. The habitat of the Italian locust spreads across Europe, Russia, Central Asia and China [58]. The different subspecies of the migratory locust such as the Asia, Oriental and African locusts are found in temperate and tropical zones of the eastern hemisphere [57]. On the contrary, the Australian plague locust, is only found in Australia. 

Most studies focused on study areas in China (26%), followed by Australia (13%), Mauritania (12%), Uzbekistan (7%) and Kazakhstan (5%). There are no studies for the Arabian Peninsula, Pakistan and only one for India, although those regions are highly vulnerable, e.g., to desert locust outbreaks. English-language publications using remote sensing for locust research or management were barely found for North and South America, South-East Asia and Europe. This may be due to minor risk of locust outbreaks (e.g., in case of Europe) or that applications use data sources apart from remote sensing, e.g., field and station measurements (e.g., in case of North America) [59,60,61].

#### 3.2.3. Sensors and Variables

**Figure 9:**
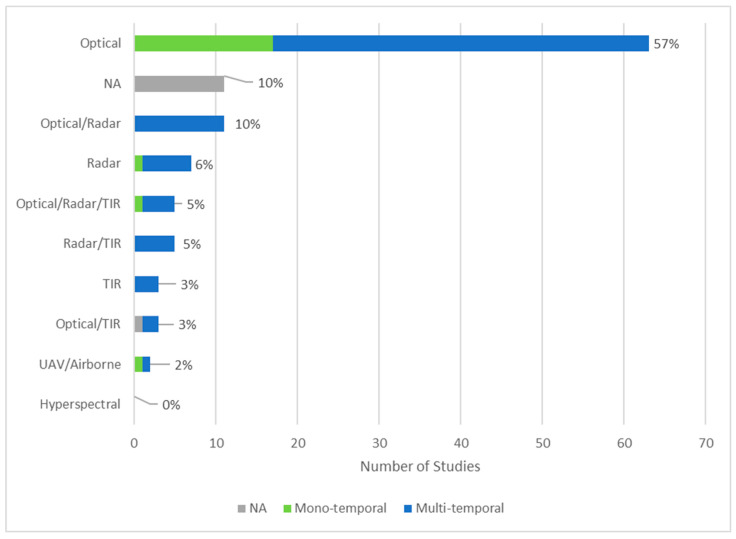
Total number of studies and remote sensing sensor types used.

In this section, we quantify the studies based on different sensor types, derived variables and metrics. The reviewed publications show a distinct dominance with 57% of using optical instruments only (Figure 9). This dominance is due to the fact that the detection of green vegetation and its density is of high importance for locust habitat monitoring as well as for damage assessment. With few exceptions the authors used data from AVHRR, MODIS, Landsat and SPOT-VGT sensors. Applications of radar sensors were found in 6% and in combination with other sensors in an additional 20% of the studies (optical/radar 10%, optical/radar/TIR 5%, radar/TIR 5%). Passive and active radar sensors are applied for soil moisture, precipitation and wind estimations. 

The category of sensors including thermal infrared (TIR) is related to temperature estimation which is, together with rainfall, important for monitoring as well as for hatching and outbreak prediction. In combination, there were 16% of studies using TIR (optical/radar/TIR 5%, radar/TIR 5%, TIR 3%, optical/TIR 3%). There were no studies using satellite-based hyperspectral sensors and only two studies (2%) referring to data from airborne and UAV cameras. 

**Figure 10:**
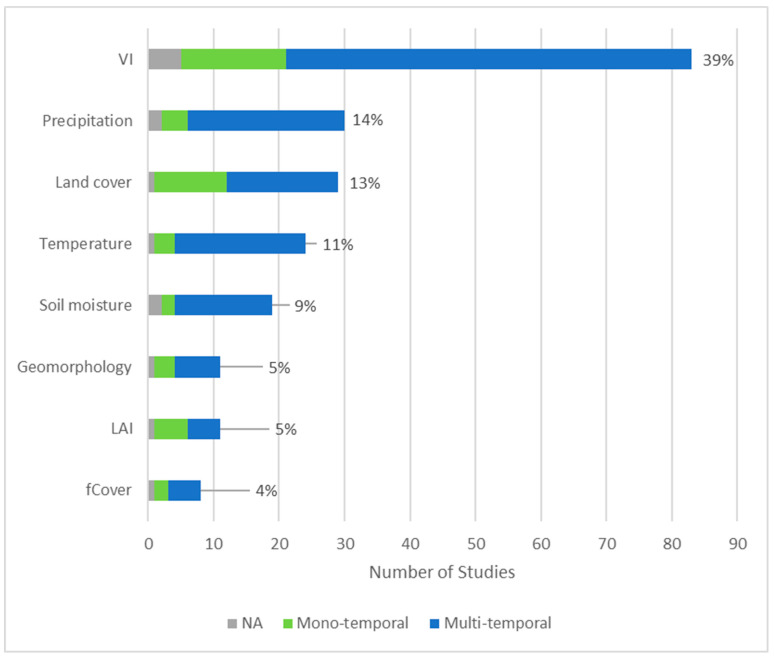
Satellite-based studies categorized into used/derived parameters/variables.

Among variables, parameters and metrics, we found that vegetation indices (39%), precipitation (14%), land cover classification (13%), temperature (11%) and soil moisture (9%) are dominant (Figure 10). Within the vegetation indices (VI), the Normalized Difference Vegetation Index (NDVI) was applied in most cases with only few exceptions (e.g., Enhanced Vegetation Index (EVI)). Furthermore, the usage of geomorphological metrics derived either from optical or SAR data have shown great potential [62] but its application was found only in 5% of studies. Moreover, very few studies use the Leaf Area Index (LAI) (5%) or fraction of vegetation Cover (fCover) (4%).

### 3.3. Thematic Foci

**Figure 11:**
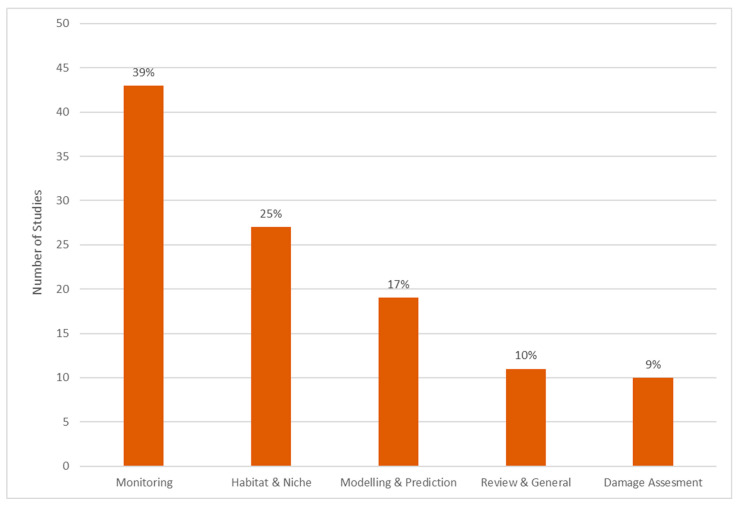
Total number of studies categorized in major research topics.

As described in the introduction, remote sensing can add valuable information at different critical time steps of the locust life cycle (Figure 3). This depends on temporal as well as on spatial scale. For example, ecological niche modeling considers species-relevant variables and are mostly applied on regional to continental scales with up to 1 km spatial resolution by utilizing long-term climate data (e.g., WorldClim [63] or National Centers for Environmental Modeling (NCEP)/National Center for Atmospheric Research (NCAR) reanalysis data [64]) and environmental variables such as soil structure or terrain. Contrary, the damage on vegetation by instar nymphs can only be assessed with high to very-high-spatial-resolution (VHR) satellite sensors with a spatial resolution of few ten meters up to centimeters. Overall, the literature review revealed five major thematic categories (Figure 11):

- Habitat mapping and ecological niche modeling as static state description of potential habitat where locust might breed.
- Habitat monitoring as temporal description focusing on variable environmental parameters relevant for locust development.
- Outbreak and hatching prediction as forecast component for future.
- Damage and loss assessment as post outbreak evaluation.
- Overarching review and general research papers.

The thematic categorization of reviewed studies was performed by examining the major objectives and presented results. If the objective of a study was to map or describe habitat or ecological niche of a locust species, it is grouped into the category “habitat mapping”. The major result can be categorical habitat maps for a certain time or time period, as well as probability assessment about which areas are more prone to locust breeding. Studies which focus on monitoring or detecting changes of ecological parameters over time are grouped in “habitat monitoring”. Here, the focus is on analyses at high temporal frequency or operational monitoring of ecological parameters which affects locust life cycle and potentially contribute to early warning. Studies focusing on forecast are grouped in “outbreak and hatching prediction”. For these three categories, there are studies which might include components in line with two or even three described categories. For example, most studies grouped into “outbreak and hatching prediction” also contain monitoring aspects because it is an important tool to predict outbreaks and many forecast approaches are constructed based on statistical relationship between historical field data and relevant ecological and meteorological parameters. In these cases, we categorize based on the most important outcome. The grouping into “damage assessment” and “review and general” was more straight forward due to none intersecting objectives.

#### 3.3.1. Habitat Mapping Studies

Identifying habitat and possible breeding sites is one of the most important tasks for implementing cost- and time effective pest control [5]. Since the introduction of Landsat and AVHRR sensors, identifying potential locust habitats has been an essential priority for locust management services to prioritize monitoring. We identified two main approaches which have been used to map, model or classify suitable habitats of locust species, (i) land cover-based habitat mapping and (ii) habitat suitability assessment or modelling-based ecological niche estimation. The most important information and outcomes are summarized in following subsections for each approach.

##### Land Cover-Based Habitat Mapping

The first approach utilizes land cover classification methods. The outcome of land cover-based mapping are usually categorical maps of land cover or vegetation classes, which also might be converted into risk or habitat suitability classes (e.g., high, middle, low). At the beginning, researchers, e.g., McCulloch and Hunter [40], classified locust habitats using Landsat MSS data at a 90 m spatial resolution by visual image interpretation. Based on expert knowledge about the ecology of different species and preferred vegetation types, habitats can be described by different land cover types. In this way, it is possible to indirectly assess the suitability for locust breeding. This strategy has been widely applied, especially for migratory locust species which breed in wetlands with reed vegetation (e.g., *Phragmites australis*). These habitats are highly dynamic in terms of inundation, which defines the locust population density and therefore triggers outbreaks. Sivanpillai et al. [65] applied unsupervised classification approach using 30 m spatial resolution Landsat images in Ili river delta (Kazakhstan) to identify land cover classes which provide favorable conditions for the Asian migratory locust. A similar strategy was used in Latchininsky et al. [66] and Sivanpillai and Latchininsky [67] for selected Landsat images in Amudarya delta (Uzbekistan). In Sivanpillai and Latchininsky [68] the authors identified common reed areas as potential Asian migratory locust habitats in Amudarya delta based on time-series analysis of MODIS 8 day NDVI composites (250 m spatial resolution) between April and September which represented the phenology of reed vegetation. In the same study region, Navratil and Wilps [69] applied an object-based classification approach using one SPOT-5 image (10 m spatial resolution) to identify reed vegetation densities and categorize them into potential habitat functions such as feeding and breeding habitats. In this way, Navratil and Wilps demonstrated the potential of higher-spatial-resolution imagery as well as segmentation-based classification methodology. Later, Löw et al. [70] analyzed MODIS EVI time series (250 m spatial resolution) between 2003 and 2014 to derive land cover for the entire Amudarya delta and relating it to migratory locust breeding sites. In this study, the authors utilize annual temporal signature to achieve high classification accuracy for each year. The classification results are finally used to derive potential risk categories and in this way support locust management. 

Additionally, research efforts on habitat mapping have been conducted for the migratory locust in several study sites in China. Q. Liu et al. [71] applied land cover classification-based approach to derive potential habitats in Yellow River delta based on one Landsat TM (Thematic Mapper) image. Li et al. [72] used 14 HJ-1 CCD images (30 m spatial resolution) to derive NDVI time series to produce a land cover classification map and convert it to potential habitats of Asian migratory locusts in Hebei Province. Zheng et al. [73] applied decision tree-based classification for six Landsat Operational Land Imager (OLI) images in the Dongying region to derive Oriental migratory locust habitat in 2015. Shi et al. [74] analyzed time series of MODIS and Landsat data between 2000 and 2016 to estimate annual changes in Oriental migratory locust habitat. Recently, Zhao et al. [75] identified land cover and land use changes in Oriental migratory locust habitats for entire China. They classified multi-annual Landsat TM, Enhanced Thematic Mapper (ETM) composites generated from data between 1993 and 1997, 2003 and 2007 and 2015 and 2018 to compare the habitat status in the years 1995, 2005 and 2017 concluding that Oriental migratory locust habitats decreased due to the change in land use. Geng et al. [76] introduced a Patch-based Analytic Hierarchy Process (PB-AHP) and Habitat Suitability Index (HSI) model based on MODIS and Landsat time series to analyzing Oriental migratory locust habitat factors in Tianjin province that affect locust oviposition and growth. The habitat factors included vegetation coverage, land cover classification, soil moisture, soil salinity and land surface temperature. The PB-AHP model was used to derive weight coefficients for each habitat factor and the degree of patch scale suitability by quantitative analysis of landscape structure and in this way map locust habitat at different suitability levels.

On the contrary to reed vegetation for the migratory locust, the detection of plant species which are favored by other locust species is more challenging due to the spectral characteristics of most optical sensors. Therefore, studies for other locust species rather focus on the general state of vegetation as a proxy for favorable breeding or invasion areas. For example, Bryceson [43] utilized Landsat MSS data to determine the location of Australian plague locust eggbeds based on vegetation greenness as areas favorable for invasion and land cover type as areas favorable for oviposition. She concluded, however, that using only NDVI information without land cover information (e.g., woods, forest versus grassland and shrubland) remains problematic. In this context, Bryceson [43] shows a high correlation between low NDVI values (−0.13 to 0.04 range) and localized nymph bands for certain land cover types (grasses and forbs and natural pasture). De Miranda et al. [77] used Landsat images to map the static state, and AVHRR-based NDVI time series to map the dynamic development of the biotopes of one grasshopper species (*Hammatocerus schistocercoides*) in Mato Grosso, Brazil. Dreiser [78] and Voss and Dreiser [79] produced detailed habitat maps for selected pilot regions within the recession area of the desert locust in Sudan, Mali, Mauritania and Niger using Landsat data, field observations and expert knowledge. Another approach was introduced by Lazar et al. [62], who integrated 43 years of field data in combination with selected Landsat images to classify main breeding sites of the desert locust during solitary phase. Their approach focused on identifying geomorphological structures such as wadis. The results for the pilot region in southern Algerian Sahara show that wadies contained 81% of observed laid egg pods according to the field data archive. Lazar et al. [62] suggested ignoring the vegetation dynamics and focusing on correlations between breeding areas of solitary locusts and specific geomorphological features such as wadis. On the other hand, the study states also that 19% of laid eggs within the test region were outside of such areas. Therefore, such approach should be applied in combination with vegetation dynamics to account for all suitable areas. 

A unique human-locust species inter-connection example can be found by examining the Italian locust. The Italian locust prefers sagebrush (*Artemisia* spp.) which also grows on fallow and abandoned fields, overgrazed pastures, as well as along roads and other man made structure [58,80]. However, when crop fields are plowed, the egg pods of the Italian locust are destroyed mechanically. Therefore, land management practice and abandoned fields as well as artificial landscapes directly influence areas favorable for Italian locust breeding. In this context, Sivanpillai et al. [81] presented a case study for mapping Italian locust habitats in Northern Kazakhstan. The authors used an Advanced Wide Field Sensor (AWIFS) scene at spatial resolution of 56 m to discriminate active and abandoned fields to identify potential breeding areas. Furthermore, Liao et al. [82] investigated three critical development stages for the Italian locust relevant to locust density—breeding stage, incubation stage and development stage—to assess a risk index in Xinjiang, China. The authors identified soil texture, vegetation species and geographic elevation as relatively temporal static geophysical properties and combined them with dynamic soil moisture, vegetation coverage, air temperature and rainfall variables. Finally, suitability index was derived for each development stage and combined to a locust plague risk index (LRI).

##### Modelling-Based Habitat Suitability Mapping

Another approach to identify habitats is based on spatial distribution models (SDM) or ecological niche models (ENM) by combining locust presence locations (derived from ground surveys) and different sets of environmental variables. ENM are usually based on machine learning algorithms to correlate a set of environmental conditions to species presence and absence records and thus predict its suitable habitats [5]. The output of such models reflects habitat suitability by fitting a probability distribution for selected species over a specific region of interest.

Aragón et al. [83] estimated climatic favorable areas for different locust species distribution and outbreaks in Spain, utilizing bioclimatic variables derived from WorldClim data and historical outbreak records. The authors tested several SDMs and summarized that temperature annual range, precipitation of the coldest annual quarter and estimated Acrididae richness had the highest influence modelling historical outbreak results. Furthermore, the authors used the Global Land Cover 2000 product (based on SPOT-4 imagery) to derive land use and assess the risk in economic important regions. Zhang et al. [84] selected key habitat factors by intersecting field data with different environmental variables such as soil properties, MODIS NDVI, geomorphological parameters derived from digital elevation model (DEM) to finally map the potential occurrence of grasshoppers (*Oedaleus decorus asiaticus*) in the Inner Mongolia steppe. Relevant climate variables influencing oviposition, overwintering and incubation were considered within a fuzzy evaluation model (multi-objective linear weighted function).

Malakhov et al. [55] pointed out that their model is able to identify areas where, at a certain time, a successful development of locust eggs is most probable, rather than to predict the actual oviposition areas. For locust management, however, the question “which areas provide favorable conditions for egg survival” is even more critical. Based on their analysis for the Asian migratory locust in Ili river delta (Kazakhstan), the ambient air temperature; the temperature of the soil during the cold season of the year, soil moisture, and the presence of reed vegetation which was classified from MODIS data were most important variables to map optimal oviposition areas. Similarly, Malakhov and Zlatanov [85] developed an ENM for the Moroccan locust combining a total of 74 variables (including satellite-based NDVI and Soil Water Index) and this way identifying favorable condition for egg pods survival. The output reveals that 58% of key variables describe winter and spring conditions, which relates to most vulnerable life stage of this species (embryogenesis and nymph development) [85].

Recently, Kimathi et al. [5] used maximum entropy model and desert locust field data to derive potential breeding areas across affected countries in East Africa. They used long-term temperature and precipitation (based on 1970–2000 data from WorldClim2) to calculate the long-term mean for December, January, February and March as well as an average soil moisture and soil sand content (at a depth of 5–15 cm). Furthermore, they included a 10 day composite vegetation greening onset product which is based on SPOT and MODIS data to assess vegetation development within modelled breeding areas. However, the authors stated that additional detailed assessment of temporal variation in vegetation prevalence and vegetation type could improve the accuracy of the model [5].

#### 3.3.2. Habitat Monitoring Studies

In the following, we summarize studies which focused on the temporal monitoring of environmental conditions, which determine the phase change as well as the timing of hatching. In this way, those studies focus on information about temporal dynamics rather than a static habitat status or potential species distribution as described in the previous section. Another main difference to previous section is that following studies potentially contribute to operative service or enable immediate decisions as part of early warning system (e.g., sending field teams for on ground monitoring or control measurements). The majority of habitat monitoring studies were focusing on precipitation and soil moisture monitoring as well as assessing vegetation change.

Early research conducted by Cherlet et al., Hielkema et al., Hielkema, Hielkema and Snijders, Tucker et al. [31,32,45,86,87] discussed different approaches on how Meteosat or AVHRR data can be utilized for monitoring desert locust habitats especially during recession periods as well as for the Senegalese grasshopper [46,47,48]. The geostationary Meteosat satellites provides data to monitor weather system over large areas at very high frequency. The identification of “cold” rain-bearing clouds, based on threshold approach in thermal infrared (TIR) channel, enables the location of areas where sufficient rainfalls and soil moisture can lead to egg hatching [86,88]. In Hielkema et al. [87] the potential breeding activity factor (PBAF) was introduced as a function of amount of pixels for four different NDVI ranges. Based on these research, remote sensing applications were implemented into FAO monitoring systems (Africa Real Time Environmental Monitoring Information System (ARTEMIS)) and build the base for instructions and guidance for national and regional desert locust management offices in affected countries. In this context, the estimation of precipitation has been the main aspect for locust and grasshopper monitoring. Dinku et al. [89] evaluated and compared seven different satellite-based rainfall detection products, which are based on thermal infrared (TIR) observations and long microwave (LM) rainfall estimation. The authors concluded, that in arid and semi-arid areas, a significant overestimation of rainfall occurrences turned out as the main weakness. Nowadays, 24-h, 10 days and monthly rainfall cumulative products which are generated by Climate Prediction Center MORPHing (CMORPH) algorithm are used for operative monitoring [90].

Recent research to monitor (i) vegetation, (ii) soil moisture, and (iii) studies which investigate combination of several ecological import variables are summarized in following three subsections.

##### Monitoring Vegetation Change

In the last 15 years, there was increased development in monitoring vegetation. Major focus was placed on temporal scale and relation of vegetation indices variability to locust development.

Ceccato [91] combined 10 day NDVI composites at 1 km spatial resolution from SPOT-VGT with spectral bands to analyze favorable conditions of the desert locust for reproduction and development. They discussed the issues of significant commission and omission errors critically and recommended to add selected spectral bands (e.g., RED, NIR, SWIR) to reduce the commission error or to add MODIS data to detect sparse vegetation, which was omitted due to coarser spatial resolution of SPOT-VGT NDVI data. Furthermore, Ceccato et al. [92] presented useful applications of decadal rainfall satellite products and MODIS 16 day NDVI data to monitor the climate variability and its integration into early warning systems for desert locust management.

Tratalos and Cheke [93] found that in arid regions, coarse-scale NDVI rather correlates with precipitation than with locust population. Chen and Li [94] analyzed LAI derived from Landsat images and presence of the Oriental migratory locust and stated a significant linear relationship between LAI and the occurrence of locust density.

In Pekel et al. [95], the authors addressed the previously stated issues with high omission and commission errors in arid regions and developed a more reliable multi-temporal approach based on MODIS data and a colorimetric transformation to identify vegetated areas in near real time. The color transformation projects the red, green, blue (RGB) bands to hue, saturation and value (HSV) where hue appears as a qualitative spectral index, and its temporal variations can be interpreted as land cover change. Cressman [13] reported that the technology for green vegetation estimation is useful and accurate in terms of operation and usability in early warning system for desert locust monitoring. There, the operational use of NDVI and EVI 16 day composites from MODIS data seems to provide sufficient information to detect changes in ecological conditions, specifically greening and drying vegetation. Cressman [13] also referred to a color space-transformed HSV product developed by Pekel et al. [95], which is able to mirror the development of vegetation; moreover, he pointed out that 11 periods of 10 day composites correspond roughly to the length of one desert locust generation. The Pekel et al. [95] approach is also used operationally for FAO early warning systems and daily locust control activities. Waldner et al. [96] assessed the accuracy of the dynamic greenness maps and revealed a high accuracy in summer breeding areas of the desert locust (F-score of 0.64 to 0.87); however, they are less accurate in winter breeding areas (F-score of 0.28 to 0.40). Furthermore, the accuracy of the product depends on landscape fragmentation (R^2^ = 0.9). Therefore, the MODIS spatial resolution is still too coarse to resolve complex landscape patterns, which were responsible for 60% of the error [96]. In this context, Waldner et al. [96] further compared PROBA-V 100 m resolution data and found that the higher spatial resolution lowers the resolution bias in fragmented areas by 20% and increases the quality of the vegetation classification. Finally, Renier et al. [97] tested the hypothesis that a reliable discrimination of the onset of vegetation senescence can be achieved by jointly implementing temporal NDVI trajectories and the Normalized Difference Tillage Index (NDTI), which is sensitive to both green and dry vegetation. The authors used MODIS SWIR band, which has shown to be effective to monitor dry vegetation. Based on these two indices, the authors calculated eleven different metrics, which should represent three phenological classes “growth”, “density reduction” and “drying”. In Mauritania, MODIS 10 day composites were applied to identify onset of drying as an indicator that a habitat becomes less attractive to the desert locust. The authors further state that higher spatial resolution may play a crucial role to improve vegetation classification in arid and fragmented areas.

Additionally, Deveson [98] reported that for the APLC model, using the relative NDVI (r-NDVI) showed significant positive relationship between one-month change in r-NDVI and the presence of nymphs and nymph density for the Australian plague locust. Additionally, Wang [99] quantitatively assessed that greening of Australian plague locust habitat is related to locust appearance and population density.

##### Monitoring Soil Moisture

Soil moisture plays a crucial role for locust development. Early studies on soil moisture showed its potential, but also the restrictions of applying satellite-based radar data to operational services due to low spatial and temporal resolution [100]. Liu et al. [101] presented an approach exploiting MODIS-based soil moisture and its relationship with Oriental migratory locust plagues. They found that the soil moisture content was lower during a severe outbreak period. Moreover, they concluded that the severe outbreak was clearly impacted by reduced soil moisture during locust oviposition and incubation periods.

Escorihuela et al. [51] presented a first attempt to implement soil moisture products within operative desert locust management tools. Different user requirements and soil moisture algorithms were assessed to produce a soil moisture product at 1 km spatial resolution. Furthermore, they present an innovative approach to derive soil moisture at 100 m spatial resolution by synergizing Sentinel-1 with Soil Moisture and Ocean Salinity (SMOS) data. Gómez et al. [53] investigated the relation between desert locust presence during the solitarious phase and soil moisture conditions based on European Space Agency (ESA) Climate Change Initiative (CCI) soil moisture product (spatial resolution 0.25°). The authors analyzed the relation between the presence of the desert locust and soil moisture change for different time intervals before the date of sighting. In conclusion, the shorter time intervals of six days performed the best result and indicating that most important time interval was between 95 and 72 days before desert locust nymph presence was detected in the field. 

##### Monitoring of Several Variables

In this subsection, we summarize studies which presented monitoring strategies combining several variables of importance. Han et al. [102] presented a remote sensing-based model including LST, soil moisture, NDVI, fCover, and LAI for monitoring the East Asian migratory locust based on three different locust life cycle stages. Similarly, Gornyy et al. [103] stated that satellite monitoring enables the monitoring of ecosystem state as well as locust population. They investigated several land surface characteristics such as heat flow, evaporation rate and NDVI from AVHRR and MODIS data in relation with Italian locust density based on the fact that daily averaged evaporation rate of surface depends on the moisture supply on ground and on the possibility of vegetation to evaporate water. For the test region of southern part of West Siberia, the authors concluded that with higher soil moisture the locust population was less dense.

Another alternative monitoring approach was presented by Propastin [104,105] combining radar altimetry measurements with NDVI data (AVHRR and SPOT-VGT) to monitor the habitat of the migratory locust in Ili river, Kazakhstan. In these studies, the author found that the water level of lakes and rivers, which can be derived via radar altimetry, directly affect the distribution of common reed vegetation which influences potential habitats as well as areas for infestation.

Li et al. [106] presented a design for GIS-based monitoring and control for the migratory locust in China which also includes processing of NDVI, soil moisture and emissivity time series from MODIS data. Latchininsky et al. [107] presented different remote sensing-based applications to monitor the red locust in Madagascar using SPOT-4 and DEM data, the migratory locust in Amudarya river delta using Landsat data and the desert locust in Mauritania using MODIS data.

Gómez et al. [52] applied different machine learning approaches to create a species distribution model by integrating six environmental variables from two sensors: MODIS-based NDVI and land surface temperature (LST) as well as Soil Moisture Active Passive (SMAP)-based soil moisture root zone, surface soil moisture, LAI and surface temperature data. Based on these variables in combination with locust presence field data, the authors modelled breeding suitability for the solitary desert locust. Within their analyses the authors identified surface temperature retrieved from SMAP as most important parameter. On the contrary, MODIS LST was not as relevant. Gómez et al. [52] point out that for monitoring the time of temperature retrieval is crucial in semi-arid and arid regions with high day-night temperature range and explain the different performance for same physical variable from two different sources. In conclusion, the most relevant variables were surface temperature, NDVI, soil moisture at root zone under different time scenarios. By including all six environmental variables, the authors obtained high predictive performance (Kappa = 0.901; ROC = 0.986).

Chen et al. [108] used multiple satellite-based datasets (NDVI, LAI, soil moisture, rain fall between 2005 and 2020 and distribution to simulate potential geographic distribution of the desert locust for Africa, Asia and Europe for different months. They coclosed that LST (27.02%) and LAI (25.63%) were the main contributors to explain the achieved distribution results. Surprisingly, soil moisture was the weakest explanatory variable (2.7%). Recently, Wang et al. [109] assessed whether China is also prone to desert locust invasion during the 2020 outbreak in East Africa, India and Pakistan. The authors, identified potential desert locust habitats in China by applying simple long-term thresholds for precipitation and temperature. Afterwards, they modelled windborne movements of the desert locust to those identified potential habitats based on historical wind characteristics at different altitudes, concluding that significant invasion of potential habitats in China is very unlikely.

#### 3.3.3. Outbreak and Hatching Prediction Studies

In this section, we focus on studies which specifically target prediction of locust outbreaks or the beginning of hatching. Compared to monitoring studies from previous sections, the focus is on the future, although historical data, past measurements and monitoring are essential part of those studies. According to Rosenberg [110], the focus of locust forecast has shifted from population dynamic-based prediction of swarm development and movement towards identification of rainfall and vegetation change that initiate the growth of existing locust populations and therefore may indicate beginning upsurges and plagues. Rosenberg [110] reported that for locust forecast there are three main scales to be considered: the long-term forecast with up to 12 months is based on climate, historical data, derived anomalies and pest frequencies. One example is the FAO SWARMS (Schistocerca WArning Management System) which contains historical data back to 1930 and enables large-scale analysis for the entire desert locust distribution areas. The medium- to short-term forecast with 1–2 months and 1–2 days are handled at a national scale, e.g., operating RAMSES (Reconnaissance And Management System for the Environment of Schistocerca) where different months can be compared with previous months and same months of other years [110].

First of all, Healey et al. [111] introduced the requirements for a GIS to support desert locust operational forecasting and monitoring. The authors underlined the importance and further implementation of weather and habitat data derived from remote sensing sources. Burt et al. [112,113] proposed the usage of Meteosat IR data to estimate rainfall from cloud temperature and support forecasting early season outbreak of the Senegalese grasshopper in West Africa. The authors conclude that this approach enables to spot areas of sufficient wetting, where the Senegalese grasshopper might hatch after 2–3 weeks.

Todd et al. [114] analyzed the impact of climate variability on brown locust outbreaks in southern Africa by implementing historical climate data. Brown locust outbreaks were associated with increased rainfall in December which is also related to La Nina events. Their results suggested that there is considerable scope for future development of models for the seasonal prediction of brown locust activity in which high-frequency variability is related to climatic indices [114]. Ma and Dai [115] utilized MODIS data including NDVI, LAI, soil moisture, LST and fCover within a Bayesian prediction network to forecast the evolution of these variables, which are responsible for Asian migratory locust outbreaks. Ceccato et al. [116] analyzed the desert locust outbreak in 2003/2004 in West Africa and accompanying circumstances which favored the outbreak. They used rainfall predictions to forecast the risk of future desert locust outbreaks. Within their study, Ceccato et al. [116] also reviewed the desert locust early warning system, and assessed the feasibility of new climate prediction methods to support forecasting desert locust life cycle development and locust movements. Here, the FAO SWARMS operates on a daily basis using RAMSES ground information, meteorological data and remotely sensed images (NDVI from SPOT-VGT at 1 km and MODIS at 250 m spatial resolution for monitoring vegetation development) to conduct short- and medium-term forecasts indicating potential locust migrations and breeding areas. Additionally, the International Research Institute for Climate and Society (IRI) is forecasting environmental conditions for desert locust development to accurately predict preferable conditions, and in this way increase the response time for further reaction and preparation of controlling steps if required. IRI specifically focuses on long-term prediction of rainfall, because it is critical to the locust outbreak forecast. In this context, Ceccato et al. [116] also discussed that seasonal prediction of rainfall in North Africa is less clear due to the midlatitude storms, whose frequency and intensity are unpredictable. Long-term rainfall forecast results can be improved where oceanic conditions in the atmospheric circulation evolve relatively slowly.

Vallebona et al. [117] analyzed connections between large-scale climatic patterns and desert locust upsurges in West Africa between 1979 and 2005 using NCEP-DOE Reanalysis 2 data at monthly resolution and 2.5° grid cells as well as desert locust population dynamics from multiple sources.

Piou et al. [118] presented a forecast method coupling historical field survey and NDVI data (MOD13Q1 NDVI 16 day 250 m product) to analyze the influence of vegetation change within desert locust habitat in Mauritania. They smoothed the NDVI time series with Savitzky-Golay filter and derived in total 27 spatial and temporal vegetation metrics before the date of observation. NDVI values were extracted for different time intervals before field survey timing (16 days, 32 days, 48 days). The authors used logistic regression model to assess the relationship between all metrics and ground control points. Their analysis showed that temporal changes of NDVI between 32 and 48 days before a locust occurrence, provided the best prediction results. The results indicated that metrics describing vegetation change allow prediction of locust presence during remission periods. At local scale, Piou et al. [118] identified a non-linear relationship between mean vegetation quantity and presence of the desert locust, even if they did not consider geomorphologic variables, which plays important role for breeding sites of the desert locust (e.g., wadis and areas with water accumulation). However, the maximum NDVI followed the topographical structures. Therefore, Piou et al. [118] argued that locust population development follows vegetation development; they also state that rainfall, the time lag between the observed vegetation changes and locust presence is critical for locust prediction. The authors summarized, that tools transforming NDVI maps to predictive presence/absence maps are required to improve locust management.

Cressman [13] presented an overview for the role of remote sensing in FAO early warning systems for the desert locust which are conducted in collaboration with national locust management organizations. The DLIS constantly monitors weather, habitat conditions and desert locust population in recession areas. This holistic observation is further used to assess the current situation and to predict the locust developments. Nevertheless, Cressman [13] stated that the spatial resolution and sensor characteristics of implemented MODIS data limit the detection of sparse vegetation that is critical for locust survival and reproduction.

For the Italian locust, Tronin et al. [119] introduced the locust hazard index (LHI), which is a linear combination between NDVI, an aridity index, and the number of sunspots. The authors also investigated LST and precipitation and concluded that there was a significant relation between droughts in 1986–1991 and 1996–2000 and Italian locust outbreaks in 1988–1991 and 1999–2001 in the Siberian study region. For both periods the LHI showed good results and therefore could be potentially used as a prediction tool. Following this conclusion, Tronin et al. [119] suggested a threshold for the LHI to assess Italian locust outbreaks in the Siberian study region. In contrast, LHI did not provide reliable results for the European study region. The prediction reliability for both regions was assessed based on false alarms and missed outbreaks. They concluded that LHI did not perform well for European study region due to the larger size and its diverse landscapes, biomes and meteorological conditions.

For eastern Australia, Veran et al. [120] used MODIS data to estimate different proportions of woody and herbaceous vegetation, together with temperature and precipitation to model the spatial-temporal dynamics of the Australian plague locust. The spatial variability of outbreaks was best explained by rainfall and land cover predictors across eastern Australia. Furthermore, the authors summarized that their results show an improvement for locust outbreak forecast by implementing key environmental factors and migration in hierarchical spatial models. Zheng et al. [121] introduced a GIS-based prediction model including monthly average temperature, monthly relative humidity, elevation, slope, NDVI (from SPOT-VGT) and soil PH data for Xinjiang province, China. They reached satisfying forecast results with a multi-criteria analysis (MCA). Weiss [37] conducted detailed research on relationship between Australian plague locust adult abundance and greenness derived from MODIS-based vegetation indices composites (8 day GPP, 8 day FPAR, 16 day NDVI) at 1 km spatial resolution. Applying a Bayesian hierarchical analysis, he concluded that all vegetation indices were weak predictors for adult locusts and investigated time period between 2000 and 2009 and therefore were no link between pests and vegetative conditions. In Mangeon et al. [122], the authors present statistical model approaches using Generalized Linear Models (GLM) and Generalized Additive Models (GAM) to quantify relative strength of different variables influencing Australian plague locust population and estimate locust abundance. Their results indicate divergent relationship for NDVI with adults and nymphs. The prediction performance was best for nymphs (R² = 0.461) underlining the local environment dependence of this life stage [122].

Apart from using rainfall and vegetation as variables for locust forecast, soil moisture is another critical variable to be considered. For brown locust life cycle modelling, Crooks and Cheke [123] assessed the usability of C-band SAR data (from RadarSat and ERS-2) for soil moisture retrieval as an alternative to rainfall estimation. They summarized that future application of SAR images will depend on the feasibility to acquire data on a spatial and temporal scale that is useful for forecasters. Meynard et al. [56] analyzed ecological niche differences between South and North desert locust subspecies during the solitarious phase and possible future shifts in geographical distribution based on climate change scenarios. Using a set of SDMs and climate variables, the authors concluded strong niche conservatism between both subspecies. Piou et al. [54] investigated temporal development for NDVI, soil moisture, rainfall and land surface temperature around survey points of desert locust presence in recession areas. The authors applied statistical analysis for all variables separately to assess their individual potential to explain and forecast desert locust presence. In this context, NDVI was the best explanatory variable (Area under the receiver operating characteristic curve (AUC) = 0.7264), followed by soil moisture (AUC = 0.6280), LST (AUC = 0.6201) and rainfall (AUC = 0.5797). In terms of vegetation response, the period of 0–48 days was found to be most important after NDVI value reaches 0.14 or higher. Additionally, very low NDVI values (below 0,10) between 160 and 80 days before locust presence, was also important. Furthermore, the analyses revealed higher chances to find locust nymphs 70 days after soil moisture increased over a period of 20 days (above 0.09 cm^3^/cm^3^) and followed by consecutive decrease. Hereafter, the random-forest forecast model combining soil moisture data with NDVI showed promising results with high AUC value of 0.761 and out of the box error of 23.7%. The model validation for years between 2010 and 2016 reached AUC between 0.583 and 0.709 and error between 27.6% and 39.7%.

#### 3.3.4. Damage and Loss Assessment Studies

Stressed or damaged vegetation is characterized by a difference in reflectance compared to healthy vegetation. Due to loss of chlorophyll stressed vegetation can be detected in red edge spectrum. Extreme loss of green vegetation is visible in VI (change in spectral reflectance) as well as in high-resolution SAR (change in canopy cover and structure). Studies focusing on damage assessment were conducted mainly for migratory locusts in China. These studies assessed vegetation patterns before and after a specific outbreak and thus identified affected areas. The information on whether there is a causal relation between damaged vegetation and locust swarms was mostly based on a priori knowledge and assumptions of the authors that no other factors contributed to the damage. All following reviewed vegetation damage studies can be considered as case studies at local scale and therefore with limited spatial coverage. For the East Asian migratory plague locust, Ma et al. [124,125] performed a calibration and verification study for Landsat data to detect damage in reed habitats. In their experimental study, Ma et al. [125] investigated whether field measurements of biomass and LAI and Landsat-based NDVI/ARVI (Atmospherically Resistant Vegetation Index) are related during locust presence (R^2^ = 0.6474). Ji et al. [126] used MODIS NDVI time series to assess damage due to an Oriental migratory locust outbreak in Hebei Province, China. Zha et al. [127] analyzed MODIS-based multi-spectral indices using temporal filtering and concluded that NDVI was the best index to assess damages caused by locust outbreaks. Liu et al. [128] and Tian et al. [129] calculated Landsat-based NDVI difference maps to assess the differences before and after outbreak event. With the focus on vegetation loss, Zha et al. [130] introduced the Locust Density Index (LDI) which considers the initial state of vegetation as well as the destroyed vegetation after infestation. Singh et al. [131] conducted measurements with a ground-based X-band Radar to assess the damage by *Heiroglyphus nigrorepletus* on sorghum. Furthermore, Song et al. [132] estimated reed loss caused by the migratory locust using UAV-based data.

Weiss [37] also investigated the capacity of MODIS 1 km temporal composite products to map vegetation damage caused by nymph bands of the Australian plague locust. The extensive statistical analyzes between prior, during and post presence of bands showed no significant relation to area extent or intensity of damaged vegetation. In conclusion, Weiss stated that coarse spatial and spectral resolution as well as temporal compositing methodology of used products were the main reason why vegetation damage caused by nymph bands feeding was not detected.

Additional to satellite-based studies it is interesting to note that Hunter et al. [38] analyzed Australian plague locust bands which were observed from an airplane. There, the accumulation of locust nymphs as well as damaged vegetation is clearly visible in RGB images. VHR satellite data (e.g., WorldView-3, GeoEye, SuperView) as well as data from UAV and very-high-spatial-resolution sensors should be capable of spatially resolving such accumulation of locusts and damaged vegetation. 

#### 3.3.5. Review and General Studies

In our literature search, we found six review and four general discussion publications dealing with locust pests and remote sensing applications. Cracknell [133] discussed general capacities of remote sensing detecting habitat changes and applicability for locust management. Hunter [34] presented APLC activities and demonstrated that Australian plague locust bands can be spotted using airborne imagery with spatial resolutions similar to today’s VHR satellites. Maiga et al. [20] review paper focused specifically on the ecology and management of the Senegalese grasshopper. The authors summarized also the potential of remote sensing and encouraging results for the Senegalese grasshopper from early studies on AVHRR NDVI and Meteosat IR data which demonstrated that suitable breeding areas can be identified with simple thresholding methods.

Latchininsky and Sivanpillai [57] presented an overview of existing EO sensors, their spatial and temporal scales as well the potential of GIS technologies for locust monitoring and risk assessment to promote these technologies for further usage. Further, Latchininsky [58] gave a comprehensive state-of-art review showing that in 2013 most operative applications were conducted by FAO and APLC, focusing on vegetation and meteorological parameters. Additionally, Latchininsky [58] provided details for other destructive locust species, their ecology and EO applications for their monitoring.

Huang [2] provided a review on EO application for locust and grasshopper plagues specifically in China focusing more on ongoing research in monitoring as well as risk and loss assessment. For risk assessment, Huang [2] summarized that habitat mapping by multi-spectral land cover classification (Landsat, ASTER, HJ-1 CCD) was dominant. For monitoring, studies focused mostly on vegetation (MODIS time series), soil moisture and land surface temperature with high temporal resolution due to rapid changes of these critical variables.

The review paper of Zhang et al. [4] covered control measurements and locust ecology but also paid attention to EO as an important tool in modern locust management. This review provides a comprehensive overview of different locust species, historical outbreaks and existing locust and grasshopper operational management systems. Zhang et al. [4] concluded that the knowledge about locust biology, ecology and the interaction with human-made effects promoting outbreaks of locusts and grasshoppers must be improved; in this way, new and improved methods to forecast and monitor gregarious locust infestations are required.

Recently, Abd El-Ghany et al. [134] published a review dealing with EO application as a promising strategy for insect pests and diseases management. This review provides a short technical overview of EO sensors and their potential to detect and monitor different insects and agricultural pests.

## 4. Discussion

### 4.1. Contribution of Remote Sensing to Locust Management

In this section, we reflect on the main remote sensing contribution for improved locust management and recent trends. First of all, in regards to habitat mapping, recent approach has been shifted from single image land cover analyses [43,65,79] towards implementing time series-based classification to generate results for different time steps and thus enable long term habitat and species distribution quantification [70,75].

Secondly, in terms of habitat monitoring, there was a district development. In 1991, Cracknell [133] discussed that the prospect of direct detection of habitats changes are unrealistic or only possible with considerable time lag. In 2002, Crooks and Archer [100] summarized that soil moisture dataset were not available or restricted to be used on operative base. Looking at the progress in 2008, Maiga et al. [20] stated that the link between acridian risk and monitored ecological conditions was still relatively empirical at that time. Recent progress in satellite imagery and availability of new datasets in combination with advances in methodological approaches and computing power are about to overcome those restrictions and contribute to a new era in remote sensing-based locust management: using multiple variables at higher temporal resolution and increasing spatial resolution. The introduction of MODIS data and thereafter increase in spatial resolution (250–1000 m), spectral resolution (36 channels) while containing high temporal frequency (daily) and covering large areas contributed to a major boost and improvement in locust management. Since then remote sensing-based research focused on temporal scale and statistical relation of locust occurrence and prior conditions [95,96,97,118]. The observation of vegetation change (greenness maps) over time is one of the most important application in desert locust management [13,107,118]. According to Piou et al. [118], especially coherent construction of secondary metrics derived from NDVI time series provides good prediction of desert locust presence and in this way allow a better planning of field surveys [107]. Furthermore, based on MODIS data, additional vegetation parameters (e.g., EVI, GPP, FPAR, LAI) and variables (e.g., LST) and well established Analysis-Ready Data (ARD) are provided which have enabled investigation on several important ecological variables and their relation to locust presence. Since then, together with improvement of rainfall estimation and weather prediction, this has been main remote sensing-based components for operative monitoring, early warning and prediction.

Moreover, applications of remote sensing-based soil moisture data has been comparably rare despite the acknowledged fact that it is one of the most important variables defining the survival of locust eggs as well as for the timing of hatching. In 2014, Crooks and Cheke [123] stated that application of SAR imagery in brown locust forecasting depends on reasonable access to data and useful spatial and temporal resolution for forecasters. In recent years, the addition of soil moisture datasets has been possible due to progress in SAR technology and improved soil moisture algorithms. Recently, Gómez et al. [52] published a promising approach stating the importance of soil moisture data. The future usage of 1 km soil moisture products in desert locust early warning system at national locust centers and at DLIS-FAO for the entire recession area of the desert locust (0–40 N/20 W-80 E) was introduced by Escorihuela et al. [51]. Additionally, Piou et al. [54] suggested that soil moisture shall become standard tool for preventive locust management. However, for species with very short incubation time, such as the desert locust, the availability of such datasets needs to be provided in near real time (NRT) to enable appropriate analysis and following measures. This is a challenging task especially regarding the vast areas to be monitored.

In terms of prediction, recent progress utilizes machine learning approaches and establishes statistical relationship between all available and important variables [52,54,56]. For preventive locust management, forecasting models have to be quickly updated with new satellite data [54].

### 4.2. Potential of Higher Spatial Resolution and Temporal Coverage

Former studies using coarse satellite data stated that there was no significant relation between locust and vegetation indices. Rosenberg [110] mentioned that by using coarse-spatial-resolution data, it was not possible to identify changes in regions with very low (<5%) vegetation cover, which is typical for desert locust breeding areas. Despland et al. [135] demonstrated that at continental scale (4° spatial resolution) forecast and outbreak areas are uncorrelated and therefore, they questioned the usefulness of NDVI for desert locust prediction at such a coarse spatial resolution and due to NDVI limitation in arid areas. Tratalos and Cheke [93] could not identify any linear relationship between locust breeding areas and NDVI (from AVHRR 8 km). Those studies using NDVI at low and medium spatial resolution showed restrictions especially in semi-arid regions and highly fragmented landscapes. Studies utilizing MODIS-based VI at 250 m spatial resolution and temporal relationship proved that there was significant relationship. Nevertheless, despite the improvements introduced by MODIS some restrictions have remained as stated in Cressman, Escorihuela et al., Renier et al. and Waldner et al. [13,51,96,97]. There, the authors discuss that satellite data with higher spatial resolution will provide further improvements especially for vegetation detection in arid and semi-arid regions where fragmented vegetation leads to higher commission and omission errors when using coarse resolution data. Waldner et al. [96] demonstrated an improvement of 20% when comparing MODIS data at 250 m with PROBA-V data at 100 m spatial resolution. The potential of higher-spatial-resolution data has been shown in many other disciplines (e.g., agriculture, forestry, urban development). The utilization, e.g., of Sentinel-1 (available since 2015) and Sentinel-2 (available since 2016) data for monitoring can improve spatial scale and the detection frequency. Peer-reviewed publications which use these data sources for locust research are with one exception [51] not available. In addition, a combination of Sentinel-2 and Landsat data can improve the temporal and cloud free observation frequency. The question arises as to whether such datasets can contribute to further significant improvements. Nevertheless, in terms of locust management, one has to keep in mind the usability and feasibility for vast areas in limited resources especially in developing countries. On the one hand, a possible improvement alongside higher spatial resolution needs to be contrasted with time management, reliability and additional required resources and also justify the needs of locust managers. On the other hand, further research can demonstrate improvements and enable operation with further technical and economic development.

Furthermore, using Landsat, and eventually Sentinel data, the detection of damage assessment has been proven to be feasible. Nevertheless, economical loss assessment caused by locust plagues and outbreaks from remote sensing data is still rare [37]. The Landsat archive with data over more than 40 years offers unique opportunities to perform further long-term analysis. For example, systematic damage assessment, vegetation development and quantification related to past large-scale outbreaks can benefit from this data source, although the temporal resolution of Landsat is limited. Long-term analysis and quantification of vegetation structure dynamics as well as land cover and land use change and their relation to locust population dynamics and outbreaks are still rare (Figure 6). This fact can be related to high data costs and limited availability before satellite archives were accessible free of charge. Furthermore, locust outbreaks are irregular events and therefore, several studies mostly focus on these specific outbreak years. Nevertheless, research with long-term character is important to investigate the entire range between derived parameters and solitarious and gregarious locust presence. For example, in Tratalos and Cheke [93], the authors analyzed long NDVI time series to understand whether NDVI is related to different locust phases and population densities or rather to precipitation variability only. Therefore, additional studies covering longer time periods providing a connection between different environmental factors and locust populations might provide new insights.

Finally, the potential and possible benefits of VHR satellites are basically unexplored. Additional studies need to provide a better understanding of how VHR data can be exploited for early warning and detection of early instar activity (e.g., locust bands) and damage assessment. EO data and archives provide the required specifications to tackle these challenges and investigate benefits and restrictions.

### 4.3. Discrepancy between Research Origin and Region of Interest

**Figure 12:**
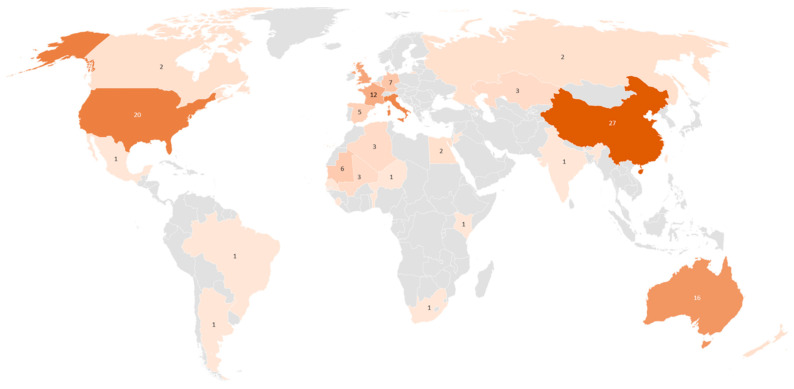
County of origin of authors’ affiliation.

The majority of publications focus on the desert locust and migratory locusts affecting large parts of the African continent, the Arabian Peninsula, India and Pakistan. The third foremost species is the Australian plague locust. For the migratory locust and the Australian plague locust, we found a clear relation between investigated regions of interest and the countries of affiliation of the authors (Figure 8 and Figure 12). However, there seems to be a gap for desert locust-affected regions as well as for other locust species. One reason is the absence of English-speaking studies despite a wide existing knowledge in affected countries. Additional research is probably available in other languages (e.g., Chinese, French, Russian, Spanish) but is less visible within the English-speaking literature. Furthermore, the absence of English-speaking scientific publications may also be due to the periodic occurrence of locust plagues combined with the fact that many countries and regions have not dealt with these challenges for several decades [26]. Moreover, as locust outbreaks are not limited by country borders, more multi-national research on EO applications would contribute to further understanding of locust–human inter-connections as well as for improvement in locust management. Another reason may be the absence of funding to promote further research because locust management is rather pragmatic with the overarching goal in an effective control of outbreaks and not academic publications. Nevertheless, there is still a lack of English-speaking, peer-reviewed literature and studies conducted by organizations or universities located in affected regions. Involving local stakeholders with their knowledge and experience, would definitely contribute to a further development and to an improved exploitation of EO capacities for locust management and research.

### 4.4. Overall Lack of Remote Sensing Application for Locust and Grasshopper Species

Despite the progress been made for the desert locust, the migratory locust and the Australian plague locust (shared total of 74%), there is a lack of studies for other pest locust species (Figure 7). This lack has been documented in previous studies and is also related to less advanced or absent organized preventive locust management [58,107]. In our review, we quantify the actual rate of conducted studies which reveals the unequal distribution for other species not only because of absence of operative management but also in conducted research. Since Latchininsky’s review in 2013 [57] the progress and investigation for other locust species remained minor. Therefore, the question arises as to whether remote sensing datasets and applications might be insufficient to map and monitor variables of importance due to more complex environment or is the lack of studies because of restricted funding or academic resources dealing with other locust species? Based on developments and encouraging results for the desert locust, the migratory locust and the Australian plague locust, research and management for other species could benefit from further remote sensing-based applications. Studies over the last four decades provide a good foundation. Nevertheless, field observations and extended species-specific ecological and biological knowledge are crucial to achieve meaningful results when applying remote sensing.

### 4.5. Potential of Alternative Methods and Analysis

Recent studies focus on comprehensive analysis of several essential ecological variables. This is due to the availability of more and more ARD. In this way, scientist can focus on the relation and individual importance of variables rather than dealing with extensive raw data preprocessing. Reviewed studies have been applying mostly NDVI to assess the risk of gregarization, to predict hatching and outbreaks, or simply use the technique as a metric for land cover classification. However, the capacity of NDVI in arid areas has been controversial. Therefore, at the background of new options and cloud computing possibilities, the benefit of additional indices or variables can be explored and compared. For example, Cherlet et al. [87] concluded that results achieved using PVI were most reliable. However, the operative usability at a large scale was not feasible at that time. Here the question arises as to whether application of other indices can provide significant improvement or not. At the background of previous discussion and findings following investigations focusing on additional strategies to prove improvements or limitations can be addressed:

- Further research on geomorphological variables for the desert locust as suggested by Lazar et al. [62].
- Application of sensor fusion/combination to minimize restrictions of sensor characteristics [136,137].
- Application of hyperspectral data to enable more detailed classification of vegetation types, stressed vegetation or damage [138,139].
- Time-series analysis focusing on phenology [140,141,142].
- Other indices and metrics, e.g., Soil Adjusted Vegetation Index (SAVI) [143] or Perpendicular Vegetation Index (PVI), which specifically consider ‘noise’ caused by soil [144]. The question is, can l other approaches or indices overcome restrictions which are observed in arid regions when using NDVI?
- As shown by Propastin [104,105] altimetry data in combination with VI show high potential for monitoring migratory locust habitats along rivers and lakes. In this context the new Surface Water and Ocean Topography (SWOT) mission as well as other altimetry datasets can contribute to further monitoring improvement for migratory locust species.
- Systematic and large-scale detection of damage and remote sensing-based economical loss assessment studies to evaluate economic impact and production loss on large scale. Remote sensing applications have received comparable little attention [37]. Red edge channel, e.g., from RapidEye satellites which was developed specifically to identify damaged or stressed vegetation could provide improved results for loss assessment of green vegetation [145]. The question is, can remote sensing-based damage assessment contribute to economic loss estimation on larger scale?
- Usage of VHR resolution imagery and machine learning approaches [146,147,148,149] to investigate the benefit in early locust damage and locust band detection. The question is, can dense locust bands be identified in VHR imagery?
- Further inclusion of remote sensing in ENM and HSI modelling, where all important static and dynamic environmental parameters are combined with species specific preferences [76,150,151,152].
- The importance and potential of UAV-based systems for locust management supporting ground teams requires standardized analysis and investigation for automatic image processing. The advantages of UAV-based monitoring are promising [153,154]. However, scientific evidence of benefits within locust management and research are still rare. Monitoring of vegetation state, damage assessment as well as monitoring of locust bands are possible fields for investigation. The question here is, how can UAV-based monitoring applications contribute to operative locust management?
- Finally, locusts and grasshoppers strongly depend on climate conditions such as temperature, precipitation and humidity [119]. Further research to analyze the influence of climate and environmental change to different locust species distributions and outbreak risk are therefore required [26].

Mentioned suggestions for further research have to prove their benefit and outline practical contribution towards locust management. Therefore, from a locust management perspective, one has to consider all important factors within operational services (e.g., internet connection, access to data and applicability, area to be monitored, reliability vs. spatial precision) and contrast it with possible improvements.

## 5. Conclusions

In this review, we provided an extensive overview of 110 English-speaking, EO-related research articles with respect to destructive locust/grasshopper pest species. On the one hand, our focus was to quantify different aspects of reviewed studies. Therefore, we categorized the studies covering (i) investigated species, (ii) areas of interest, (iii) sensor types employed, and (iv) variables used. On the other hand, we aimed to point out main research foci and reflect on the development. We categorized specific research foci, namely (A) habitat mapping, (B) habitat monitoring, (C) outbreak/hatching prediction, (D) damage and loss assessment, and (E) review and general studies. By looking at the quantified results and methodological progress, the following findings can be summarized:

- (i) Investigated species: The majority of studies focused on the desert locust (33%), the migratory locust (27%) and the Australian plague locust (14%). Remote sensing applications for other harmful locust species such as the brown locust (4%), the Central and South American locusts (1%), the Italian locust (5%), the Moroccan locust (1%) or the red locust (1%) are still very rare.
- (ii) Areas of interest: Areas of interest were mostly located in China (24%), Australia (14%) and Mauritania (11%). Despite a high risk of outbreaks from different species, there is a lack of English-speaking studies for the Arabian Peninsula (none), the Middle East and Pakistan (none), India (1%), South-East Asia (1%), North and South America (2%) and Russia (2%).
- (iii) Employed sensor types: Optical EO data were most frequently used. Here, 57% of all studies solely used optical data. Whereas, AVHRR, MODIS, SPOT-VGT and Landsat sensors were mostly employed. Following optical sensors, radar (6%) and TIR (3%) were the second and third most used sensor types, respectively. However, both were mostly applied in combination with others (optical/radar 10%, optical/radar/TIR 5%, radar/TIR 5%, optical/TIR 3%). No peer-reviewed publication was found using VHR (e.g., Quickbird, IKONOS, WorldView) or Sentinel-2 data; only one study is available using Sentinel-1 SAR data.
- (iv) Used variables: The majority of studies applied NDVI, land cover information, LAI or fCover for analysis (39%, 13%, 5%, 4%), referring to the importance of vegetation as a key parameter affecting population density and phase change of locusts. Despite the high importance of soil moisture for locust development, there are only few studies focusing on EO-based soil moisture retrieval (9%). However, recent development indicates that remote sensing-based soil moisture data will be an essential part in further research and eventually in desert locust management.
- Research foci: The majority of studies focused on habitat monitoring (39%), followed by habitat mapping (25%), outbreak/hatching prediction (17%) and general review publications (10%). Few damage assessment studies were conducted (9%); most of these studies are feasibility cases carried out for the migratory locust in selected geographic areas.
- Most articles reveal test case studies covering small study regions and short time periods. Overall, only 18 studies were long-term covering ten or more years.
- Furthermore, we found fewer English-speaking, peer-reviewed literature and studies conducted by organizations or universities located in locust-affected regions (except Australia and China).

The role of remote sensing for locust management and research has increased over the past 40 years and nowadays can be considered as irreplaceable. Well-operating monitoring and prediction systems for the desert locust (by FAO) and the Australian plague locust (by APLC) document the success and the advantage of implementing EO data to save time and resources once outbreaks occur. Summarizing, most EO applications focus on the monitoring of vegetation changes and precipitation patterns in locust habitat areas to determine potential gregarization, to stratify field surveys and to assess the risk of locust population increase [98]. In recent years, scientific attention was paid to soil moisture retrieval as well as modelling approaches combining several important variables. In terms of vegetation and land cover monitoring, the trend shows more time-series applications focusing on phenology and replace single image analysis. Overall, this review underlines further needs for EO-based research to either fully exploit the potentials of EO data and approaches or proof their limits. There is a lack of studies using available open source EO data archives over entire habitats and long time periods. Moreover, the sensors of the Sentinel fleet are still rarely applied. Here, experience from other disciplines, e.g., agriculture and forestry, may be adopted to improve results and eventually contribute to locust management. Feasibility and test case studies have played a crucial role to contribute to nowadays operative services. Applications which were unimageable a few decades ago have become operative along with technological development in terms of sensor characteristics, methodologies and computing power. Many countries launch and operate environmental and industrial satellites. Fusion and combination of available data sources might enable to detecting the Earth at very high spatial, spectral and temporal resolution. Today, the Earth is covered by VHR data from different satellites sources. Therefore, detection of locust bands might become more feasible in future. Nevertheless, extensive knowledge of considered species and geography remains a key factor in further locust-related remote sensing applications. Therefore, more inter- and multi-national research funding utilizing the full capacity of remote sensing data is required.

## Figures and Tables

**Table 1:** Categorization of research articles for this review.

Publication-Specific Aspects	Thematic Foci
Species of interest	Habitat mapping (static)
Region of interest (country level)	Habitat monitoring (temporal)
Sensor and used variables, scales	Outbreak/Hatching prediction (future)
Authors’ affiliation (country level)	Damage assessment (past)
	Review articles (general)

## Data Availability

Not applicable.
